# Necrotrophic Effector Epistasis in the *Pyrenophora tritici-repentis*-Wheat Interaction

**DOI:** 10.1371/journal.pone.0123548

**Published:** 2015-04-06

**Authors:** Viola A. Manning, Lynda M. Ciuffetti

**Affiliations:** 1 Department of Botany and Plant Pathology, Oregon State University, Corvallis, Oregon, United States of America; 2 Center for Genome Research and Biocomputing, Oregon State University, Corvallis, Oregon, United States of America; Soonchunhyang University, KOREA, REPUBLIC OF

## Abstract

*Pyrenophora tritici-repentis*, the causal agent of tan spot disease of wheat, mediates disease by the production of host-selective toxins (HST). The known toxins are recognized in an ‘inverse’ gene-for-gene manner, where each is perceived by the product of a unique locus in the host and recognition leads to disease susceptibility. Given the importance of HSTs in disease development, we would predict that the loss of any of these major pathogenicity factors would result in reduced virulence and disease development. However, after either deletion of the gene encoding the HST ToxA or, reciprocally, heterologous expression of *ToxA* in a race that does not normally produce the toxin followed by inoculation of ToxA-sensitive and insensitive wheat cultivars, we demonstrate that ToxA symptom development can be epistatic to other HST-induced symptoms. ToxA epistasis on certain ToxA-sensitive wheat cultivars leads to genotype-specific increases in total leaf area affected by disease. These data indicate a complex interplay between host responses to HSTs in some genotypes and underscore the challenge of identifying additional HSTs whose activity may be masked by other toxins. Also, through mycelial staining, we acquire preliminary evidence that ToxA may provide additional benefits to fungal growth *in planta* in the absence of its cognate recognition partner in the host.

## Introduction

Host selective toxins (HSTs), a subset of effectors produced by some necrotrophic plant pathogenic fungi, are major pathogenicity factors that mediate the disease tan spot of wheat caused by *Pyrenophora tritici-repentis* (Died.) Drechs. (syn. *P*. *trichostoma* (Fr.) Fuckel), anamorph: *Drechslera tritici-repentis* (Died.) Shoem. (syn. *Helminthosporium tritici-repentis* (Died.) or *Ptr* (reviewed in: [1,2,3,4]. Tan spot has emerged as one of the most important wheat foliar diseases in several major wheat growing areas [5,6,7,8]. The increase in disease incidence is thought to be due to the adoption of conservation tillage practices [9,10] and the acquisition through horizontal gene transfer of a potent HST, Ptr ToxA (ToxA) [11].

The importance of HSTs in pathogenicity in the *Ptr*-wheat pathosystem has been demonstrated by conversion of a nonpathogenic isolate to a pathogenic isolate through heterologous expression of genes that encode HSTs [1,12]. So far, there are two well-characterized proteinaceous HSTs, ToxA and ToxB (recently reviewed in: [1,3,4]), and one partially-characterized, non-protein HST, ToxC [13,14]. ToxA is a potent necrotizing toxin on ToxA-sensitive cultivars [15,16,17] and isolates that produce ToxA typically induce grey to tan necrotic lesions with dark centers that are sometimes associated with a chlorotic halo. ToxB and ToxC production induces chlorosis on their respective sensitive hosts, with ToxC-induced chlorosis spreading throughout the leaf [18]. The presence of other uncharacterized HSTs have been implied from inoculations on differentiating host genotypes [19,20], genetic screening [21], and partial purifications of components from crude culture filtrates [17,22]. Each of the currently identified races of the pathosystem (except for the nonpathogenic race 4) are defined by the expression of the known HSTs (ToxA, B, and C) either singly or in all possible combinations [23].

The *Ptr*-wheat pathosystem has been described as an ‘inverse’ gene-for-gene system where each individual toxin is recognized by the product of a single locus in a sensitive host and this recognition results in host susceptibility and disease [24,25]. An excellent review on the genetics of tan spot resistance in wheat has recently been published by Faris and colleagues [26]. Three independent loci in wheat have been identified that contribute to the recognition of each known HST, *Tsn1* for ToxA, *Tsc2* for ToxB, and *Tsc1* for ToxC. Sensitivity to HSTs is typically dominant, as in the case of ToxA and ToxB. The interaction between *Tsc1*-ToxC appears more complex and has been interpreted to range from recessive to dominant. *Tsn1*, which thus far is the only locus well described [27], encodes a protein similar to resistance (R) proteins that recognize ‘avirulence’ effectors [28]. Typical *Avr*-*R* gene interactions result in a ‘resistance response’ that in many cases involves a hypersensitive cell death response (HR) [29,30]. In these ‘classical’ gene-for-gene interactions the HR is associated with disease resistance but in the R gene-like *Tsn1*-ToxA interaction, the massive transcriptional re-programming, induction of defense responses, and cell death lead to disease susceptibility [31,32].

In this manuscript, we describe the impact of loss of ToxA on virulence of the *Ptr* isolate BFP (race 1) toward both ToxA-sensitive and -insensitive host genotypes. We selected BFP for this study as: 1) we have a well-assembled and annotated genome of this isolate [33], 2) there are additional HSTs produced by this isolate, i.e. the chlorosis-inducing toxin, ToxC, and unnamed proteinaceous toxins, which have been phenotypically described and partially isolated and characterized, but are currently genotypically uncharacterized [13,17], and 3) ToxA is a major pathogenicity factor in this isolate and lack of ToxA production should significantly impact virulence. We found that expression of ToxA could either positively or negatively impact symptom development dependent upon the ToxA-sensitive host genotype. In two ToxA-sensitive genotypes, ‘TAM 105’ and ‘Katepwa’, symptoms induced by expression of ToxA by BFP are epistatic to the effects of a spreading chlorosis- and additional necrosis-inducing HST. We also found that heterologous expression of ToxA in D308, a ToxC-producing (race 3) *Ptr* isolate, leads to epistasis of spreading chlorosis on ToxA-sensitive genotypes as well as increased mycelial growth in a ToxC-sensitive, ToxA-insensitive host. Data described in this manuscript support the emerging hypothesis that HSTs have an alternative function in the *Ptr*-wheat pathosystem in the absence of their cognate host-recognition partner and, further, provide the first report of necrotrophic effector epistasis.

## Materials and Methods

### Plant material and fungal isolates

The wheat cultivars used in this study include the ToxA-sensitive cultivars ‘TAM 105’[17], ‘Glenlea’ and ‘Katepwa’ [34], the tan spot resistant (insensitive to all known toxins) cultivar ‘Auburn’ [17], and the ToxC-sensitive cultivar, ‘6B365’ [34]. The *P*. *tritici-repentis* isolates used in this study were a race 1 and race 3 isolate, BFP [17] and D308 [35], respectively. BFP is a subculture derived from Pt-1C; Pt-1C was obtained from W. Bockus (Kansas State University, Manhattan, U.S.A.) and the isolate used for generation of the *P*. *tritici-repentis* reference genome [33]. D308 was obtained from L. Lamari.

### *ToxA* gene replacement, heterologous expression and transformation

The genome sequence of BFP is available for download at the Broad Institute website (http://www.broadinstitute.org/annotation/genome/pyrenophora_tritici_repentis/). Primers used for this study are presented in S1 Table. Genomic sequence data (supercontig 1.4-1447500-1450899) were used for construct and primer design to clone *ToxA* 5’- and 3’- flanking regions. These flanking regions were amplified in a mixture that contained 1.7 ng BFP gDNA, 1X GoTaq Flexi buffer, 2mM MgCl_2_, 0.2 mM dNTP, 0.25 mM each primer, and 1U Taq (Promega, Madison, WI) in a total reaction of 50 μl using the following parameters: 94°C 3 min, 29 cycles of 94°C 45 s, 58°C 30 s, 72°C 2 min, and a final extension at 72°C for 7 min. The resulting fragments were cloned into pGEM-Teasy (Promega) and identity confirmed by sequencing at the Center for Genome Research & Biocomputing, CGRB Core Facilities, Oregon State University. The 5’-flanking region was liberated by restriction digestion with *Apa*I and *Xho*I and subcloned into pCT48 [12] cut with the same restriction enzymes, just upstream of the *hygR* cassette. The 3’-flanking region was liberated by restriction digestion with *Sma*I and *Not*I and cloned into the 5’-flanking::*hygR*-containing construct cut with the same restriction enzymes just downstream of the *hygR* cassette to produce the plasmid pCVM167. pCVM167 was used as a template for PCR amplification of a single fragment or two fragments (split marker) that overlapped in the *hph* gene. PCR amplification was performed as above with 1 ng of template DNA. For transformation purposes, PCR products were purified with a Qiagen PCR purification kit (Valencia, CA). Similar PCR conditions were used for screening for homologous recombination and orientation.

Protoplasts were prepared as in [12] with modifications. One g of BFP mycelia was digested in a 25 ml flask overnight at 25°C, shaking at 60 rpm, in 9 ml osmoticum (100mM NaPO_4_, pH 5.8, 2M MgSO_4_) containing 22.5 mg Driselase (Interspex), 4.5 mg beta-glucuronidase (367,500 U/g) (Sigma-Aldrich, St. Louis, MO), and 45 mg lysing enzyme (Sigma-Aldrich) that had been filtered through a 45 μm filter. Protoplasts were filtered sequentially through 200, 100 and 50 um Nytex, collected by centrifugation, and resuspended at a concentration of 2 × 10^7^/ml STC (1.2 M sorbitol, 10 mM Tris, pH 7.5, 10 mM CaCl_2_):PEG4000 (40% polyethylene glycol (4000), 50mM Tris. pH 7.5, 50 mM CaCl_2_):DMSO (80:20:1). Transformation was performed in 15 ml round-bottom tubes on ice by adding 200 μl of protoplasts, and rolling in 2 μl 50 mM spermidine, either 3.5 μg each split-marker or 5 μg full length PCR fragment, followed by an equal amount of STC. After a 20 min incubation on ice, 200 μl, 200 μl and 800 μl of PEG4000 where rolled into the protoplasts, with a 5 min incubation on ice after each addition. After addition of 1 ml STC, the protoplasts were split into two 15 ml tubes, mixed gently with 10 ml of 46°C regeneration media (1.2 M sucrose, 0.1% yeast extract, 0.1% casein hydrosylate (enzymatic), 1.5% agar, pH 5.9), and immediately poured into a petri dish. Solidified plates were incubated at 25°C for 2 days and then overlaid with 60 μg/ml hygromycin (hyg; Research Products International Corporation, Mount Prospect, IL) in 1% water agar. Five days post overlay, colonies were transferred to V8-PDA (15% V8 juice, 10g potato dextrose agar, 3g CaCO_3_, 10g Bacto agar, in 1 liter) amended with hyg at 150 μg/ml. Preliminary screening for gene replacement was performed with the primers for *chitin synthase* and *ToxA* as described in [21]. Transformants screened for the absence of *ToxA* included all generated from the linear minimal element (LME), six from the large linear fragment, and five from the split marker.

Copy number was determined by qPCR of *hyg* with *chitin synthase A* (*CSA*, PTRG_02340) as the internal single copy gene standard. Primers were designed in primer3 (http://frodo.wi.mit.edu/primer3/) and the primer sequences tested for possible secondary structure formation in BeaconDesigner (Premier Biosoft). The possibility of amplicon secondary structure was tested with IDT scitools mFOLD. Primer efficiencies were 96 and 100% for *CSA* and *hyg*, respectively. The 1-5-1 transformant was used to generate a standard curve of Cq vs. input concentration for each amplicon. Quantitative PCR was performed with 10 ng of input gDNA and iQ SYBRGreen supermix (Bio-RAD, Hercules, CA) in the CFX96 RealTime Detection System. Data were analyzed with the CFX Manager 2.1 software package.

D308 protoplasts were produced with two times the amount of enzymes used for BFP and with 5 h incubation. Protoplasts were transformed as above with PCR fragments generated with T3 and T7 primers from the plasmids pCT48 and pCT53 [12] as described above.

### Isolate growth comparison and plant inoculation

For isolate growth comparisons, similar-sized plugs (#2 cork borer) from BFP, D308, and their transformants were grown on V8-PDA for 6 days in the dark at 25°C. Plates were scanned on an Epson Expression 1600 scanner (Epson, Long Beach, CA) at 600 dpi. For colony size comparison, colony size was estimated by multiplication of the largest and smallest diameter of the colony and mean and standard error calculated for 8 replicates. For inoculations, plugs of isolates were grown for 5 and 7 days for BFP and D308 (and their transformants), respectively. Mycelia were flooded with water (~ 8 ml), matted with a bent glass rod, incubated 24 h in constant light at 25°C, transferred to 16°C, and incubated for 24 h in constant dark. Conidia were harvested by flooding the plate with sterile E-pure water + 0.01% Tween-20, allowed to settle at 4°C, the volume reduced to ~ 5 ml, and the concentration of conidia estimated by counting in a hemocytomer (Neubauer). Two week-old plants were inoculated with 3 × 10^3^ conidia/ml Tween water. Because of the different growth rates for BFP and D308, leaves from plants inoculated with BFP and D308 (and their transformants) were harvested and scanned for documentation at 5 and 6 days, respectively. Inoculations were performed at least three times.

### DNA and protein isolation, protein gel electrophoresis and western blotting

For genomic DNA isolation, 50 ml of Difco potato dextrose broth (Becton, Dickinson, and Co., Sparks, MD) was inoculated with 5 plugs of fungal mycelia, the culture grown for 3 days at room temperature with gentle shaking (~ 80 rpm), tissue harvested on miracloth, the mycelia lyophilized, and DNA prepared with a FastDNA Spin kit (MP Biomedicals, Santa Ana, CA) following manufacturer’s recommendations. Crude culture filtrates (CCF) containing secreted proteins were produced by inoculating 50 ml of modified Fries media (15g sucrose, 5g NH_4_ tartrate, 1g NH_4_NO_3,_ 1g KH_2_PO_4_, MgSO_4_^.^7 H_2_O, 0.1g NaCl, 0.1g CaCl_2_^.^2H_2_O, 1g yeast extract in 1 liter) with 5 plugs and incubating in the growth chamber for 10–12 days. CCF was harvested through 4 layers of cheesecloth and stored at -20°C in aliquots. Protein precipitation was performed as described in [12]. Proteins were separated on a 12% SDS-polyacrylamide gel using the buffer system described in [36]. Silver staining of gels was performed with the Pierce Silver Stain kit (Thermo Scientific, Waltham, MA) and western blotting was performed as described in [37].

### Quantification of diseased leaf tissue

Leaves were scanned on an Epson Expression 1600 scanner at 600 dpi. Images were opened in Adobe Photoshop (Adobe, San Jose, CA) and an entire leaf was selected with the magic wand tool with Tolerance set to 100 and Anti-alias selected. The total number of pixels in the leaf tissue was recorded. For determining the amount of green and chlorotic tissue, the ink dropper tool was used to select either green or chorotic regions of a leaf and the color range tool was used to create a filter that represented the range of colors in each tissue type. For each leaf analyzed, the color range tool was opened, the appropriate color range file loaded, and the number of pixels containing that color range was recorded. The percent of each tissue type was calculated as (number of colored pixels/number of total pixels) x 100. The percent of necrotic tissue was calculated by subtracting the amount of percent of green and chorotic tissue from 100%.

### *In planta* mycelial staining

Leaf staining was based on the technique described in [38] with some modifications. Briefly, 1 ml 1M KOH/0.05% Silwet L-77 (Lehle Seeds, Round Rock, TX) was added to a 1 cm leaf section, tubes placed in an autoclave, the cycle started until pressure (15 psi) and temperature (121°C) was reached, and then immediately slow exhausted. The KOH was removed to new tubes for chlorophyll quantity estimation (see below) and leaf slices washed gently with 50 mM Tris pH 7.5 and equilibrated for 20 min in 10 ml 50 mM Tris pH 7.5. Buffer was removed and leaves stained overnight at room temperature in the dark with 250 μl 20 μg/ml wheat germ agglutinin labeled with fluorescein isothiocyanate (WGA-FITC; Sigma-Aldrich). After staining, a single leaf section was gently rinsed and floated into a glass Petri dish that contained 50 mM Tris, pH 7.5. The leaf section, which is very fragile at this stage, was transferred to a slide and drops of buffer added until the leaf uncurled, and then a coverslip was lightly pressed on top of the section to remove excess liquid. Leaves were imaged on a Leica MZFLIII (Bartles and Stout, Inc., Issaqua, WA) fitted with a GFP filter (Excitation 470/40nm; Barrier 525/50nm). Six fluorescent and brightfield images were captured with a CoolSnap-Pro camera (Media Cybernetics, Rockville, MD) fitted to the stereoscope and ImagePro Plus software (Media Cybernetics) and images overlaid to create a montage that approximates the entire leaf section. Leaves were also visualized at high magnification both on the fluorescent stereoscope and on a compound fluorescent microscope to ensure the low magnification images accurately reflected all of the mycelia present. At least three leaf sections from each isolate/cultivar combination where examined.

For chlorophyll estimation, the OD654 of the KOH solution that was removed from the leaf slices was measured with a Beckman DU640 spectrophotometer and total chlorophyll was estimated with the formula: μg/ml total chlorophyll = 25.7 x (Absorbance at 654 nm). A minimum of three sections per isolate/cultivar combination were examined.

## Results

### *ToxA* gene replacement in a *P*. *tritici-repentis* race 1 isolate

The loss of a major pathogenicity factor should result in an isolate with diminished virulence. To test this in *Ptr*, we targeted the gene encoding the highly-active necrosis-inducing HST, *ToxA*, for replacement. We utilized the race 1 (ToxA- and ToxC-expressing) *Ptr* isolate BFP, for which we have a reference genome, and tested several approaches for gene replacement by homologous recombination of a hygromycin resistance cassette (*hygR*) into the *ToxA*-coding region. The three approaches used included BFP protoplast transformation of: 1) a Linear Minimal Element (LME) [39] that contained a 677 bp fragment of *ToxA* fused to *hygR*, 2) a large linear fragment containing 5’ and 3’ flanking regions of *ToxA* surrounding *hygR*, and 3) two fragments of the large linear fragment described above but overlapping by 176 bp in the *hph* gene, commonly referred to as the split-marker approach [40]. After transformation, hyg-resistant transformants were selected on hyg-containing media. We obtained 26 *hygR* colonies with the LME in a single transformation, and 23 large linear and 25 split-marker *hygR* colonies total in two transformations of each. Preliminary screening for the absence of *ToxA* revealed that LME transformation had the lowest homologous recombination efficiency at 4%, whereas the large linear and split-marker approaches were much more efficient at 40 and 60%, respectively. For the experiments described in this work, we utilized colonies generated by either the large linear or split-marker approach.

**Fig 1:**
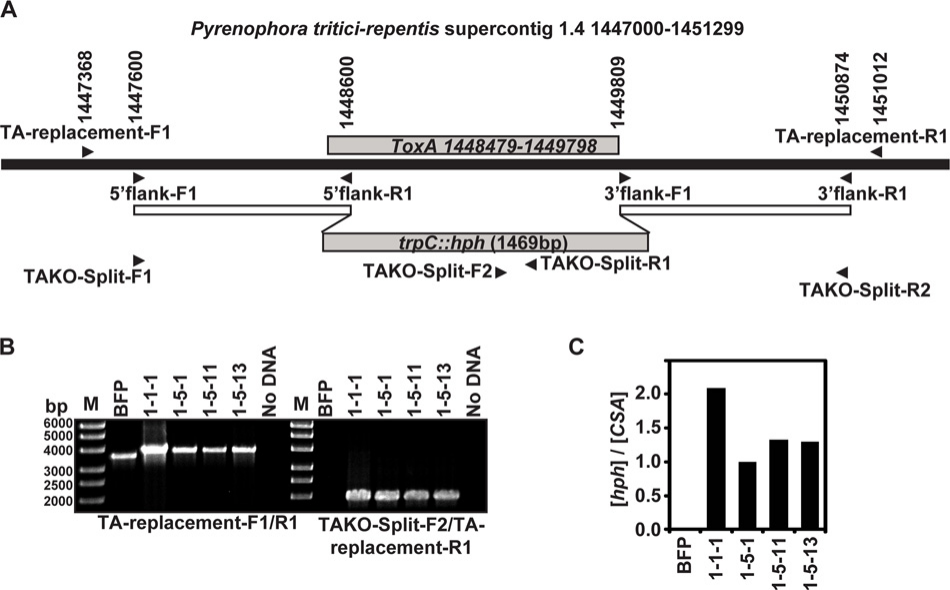
*ToxA* gene replacement with a hygromycin resistance cassette in the *P*. *tritici-repentis* isolate, BFP. (A) Schematic of the *ToxA*-containing genomic region of the *P*. *tritici-repentis* reference genome (top) and the gene replacement construct containing 5’ and 3’ *ToxA* flanking regions and the hygromycin resistance gene (*hph*) driven by the *trpC* promoter, *trpC*::*hph* (bottom). Black arrowheads indicate the position of PCR primers used for cloning and positional screening and the numbers above the top schematic indicate primer sequence position on supercontig 1.4. Genomic DNA from BFP and transformants was (B) PCR amplified to test for *ToxA* replacement (left) and proper orientation of the *trpC*::*hph* replacement construct (right) and (C), subjected to qPCR to predict the copy number of the *trpC*::*hph* fragment by calculating the ratio of the concentration of *hph* to the concentration of the single copy gene *chitin synthase A* (*CSA*). The molecular mass of standards in the molecular mass ladder (M) is indicated on the left of panel B.

To prepare the large linear and split-marker fragments, ~ 1 kb of 5’- and 3’-flanking regions of *ToxA* were first PCR amplified from BFP genomic DNA (gDNA) with primers 5’flank-F1/R1 and 3'flank-F1/R1 and cloned into a plasmid upstream and downstream of a *hygR* cassette (Fig 1A). The large linear fragment was amplified from the template with plasmid-specific primers T3/T7 and the split-marker fragments were amplified with TAKO-Split F1/R1 & F2/R2. After transformation, selection on hyg-containing media, and confirmation of the absence of *ToxA*, transformants were tested for whether the gene replacement construct was properly inserted into the *ToxA*-containing genomic region (Fig 1B). Genomic DNA of BFP and transformants was amplified with primers just outside of the putative homologous recombination region (TA-replacement-F1/R1) and separated by gel electrophoresis (Fig 1B, left panel). If the fragments had undergone homologous recombination, the PCR amplification product should be 150 bp larger than the product from untransformed BFP. All transformants tested had the larger band. To insure proper orientation, gDNA was amplified with primers that anneal to *hph* (TAKO-Split-F2) and the 3’ flanking region (TA-replacement-R1), which should generate a ~ 2-kb band only in the transformants (Fig 1B, right panel). All of the transformants had the proper sized product and as expected, no product was amplified from untransformed BFP. To confirm a single insertion, we used quantitative PCR and calculated the ratio of the concentration of *hph* and the single copy gene chitin synthase A (*CSA*), which should be approximately 1 if there is a single insertion (Fig 1C). This process estimated zero copies of *hph* in untransformed BFP, two copies in 1-1-1, and a single copy in the other three transformants, 1-5-1, 1-5-11, and 1-5-13. The two copies estimated for 1-1-1 is consistent with the increased quantity of the PCR amplification product seen when testing for gene replacement (Fig 1B, left panel). We chose the ToxA gene replacement isolates 1-5-1 and 1-5-11 for further characterization. In the remainder of the text, we refer to these transgenic isolates as *ToxA* knockouts, BFPΔ*toxA*, or by their individual isolate name.

**Fig 2:**
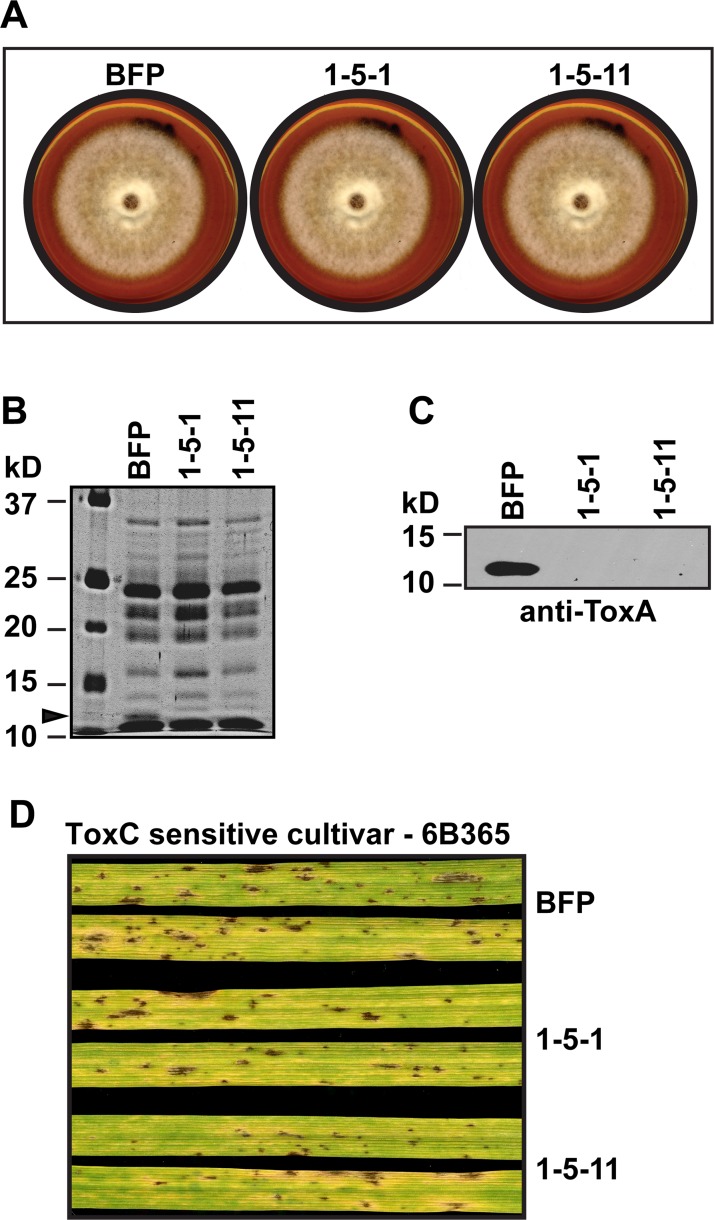
Characterization of growth and toxin production in BFP and BFPΔ*toxA* isolates. (A) Colonial morphology of isolates grown for 6 days on V8-PDA agar. SDS-PAGE of 20 μl of crude culture filtrates either (B) silver-stained or (C) on western blot with anti-ToxA antisera. The molecular mass of standards and their position are indicated on the left of each gel. The arrowhead indicates the mobility of ToxA in the BFP crude culture filtrate. (D) Inoculation of isolates on the ToxC-sensitive cultivar ‘6B365’. Leaves were harvested 5 days post inoculation.

To determine if the loss of *ToxA* or the acquisition of *hygR* altered the growth of isolates, we plated equal sized plugs of BFP, 1-5-1, and 1-5-11 on V8-PDA and documented growth 6 days after plating (Fig 2A). The two *ToxA* knockouts grew at the same rate with the same symmetric radial growth pattern as the wild type isolate, BFP. To confirm that replacement of *ToxA* with *hygR* resulted in the lack of ToxA production, we prepared crude culture filtrate (CCF) from each isolate, separated equal amounts of filtrate by SDS-PAGE, and detected proteins via silver stain (Fig 2B) and ToxA specifically by western blotting with anti-ToxA antisera (Fig 2C). The silver-stained gel showed a similar banding pattern for BFP and the *ToxA* knockouts with the exception of a band in BFP CCF at ~ 13 kDa (Fig 2B, arrowhead) consistent with the known banding pattern of ToxA [17]. Also, western blotting detected a ~ 13-kDa band only in CCF from BFP. Taken together, the SDS-PAGE results indicate that gene replacement of *ToxA* in the knockout isolates resulted in the lack of ToxA production. To determine if the lack of ToxA production impacts the production of the other known HST produced by BFP, ToxC, we inoculated the ToxA-insensitive, ToxC-sensitive cultivar ‘6B365’ and recorded symptom development 5 days post inoculation (dpi). All isolates conidiated similarly and induced dark brown lesions of varying sizes (Fig 2D). Additionally, all isolates induced similar levels of spreading chlorosis typical of ToxC-induced symptoms. This shows that the expression of ToxA, or lack thereof, does not impact the expression of ToxC, at least in this ToxC-sensitive wheat cultivar. Collectively, these data indicate that except for the production of ToxA, BFP and BFPΔ*toxA* isolates behave similarly.

### Disease reaction and *in planta* mycelial growth of BFPΔ*toxA* isolates

**Fig 3:**
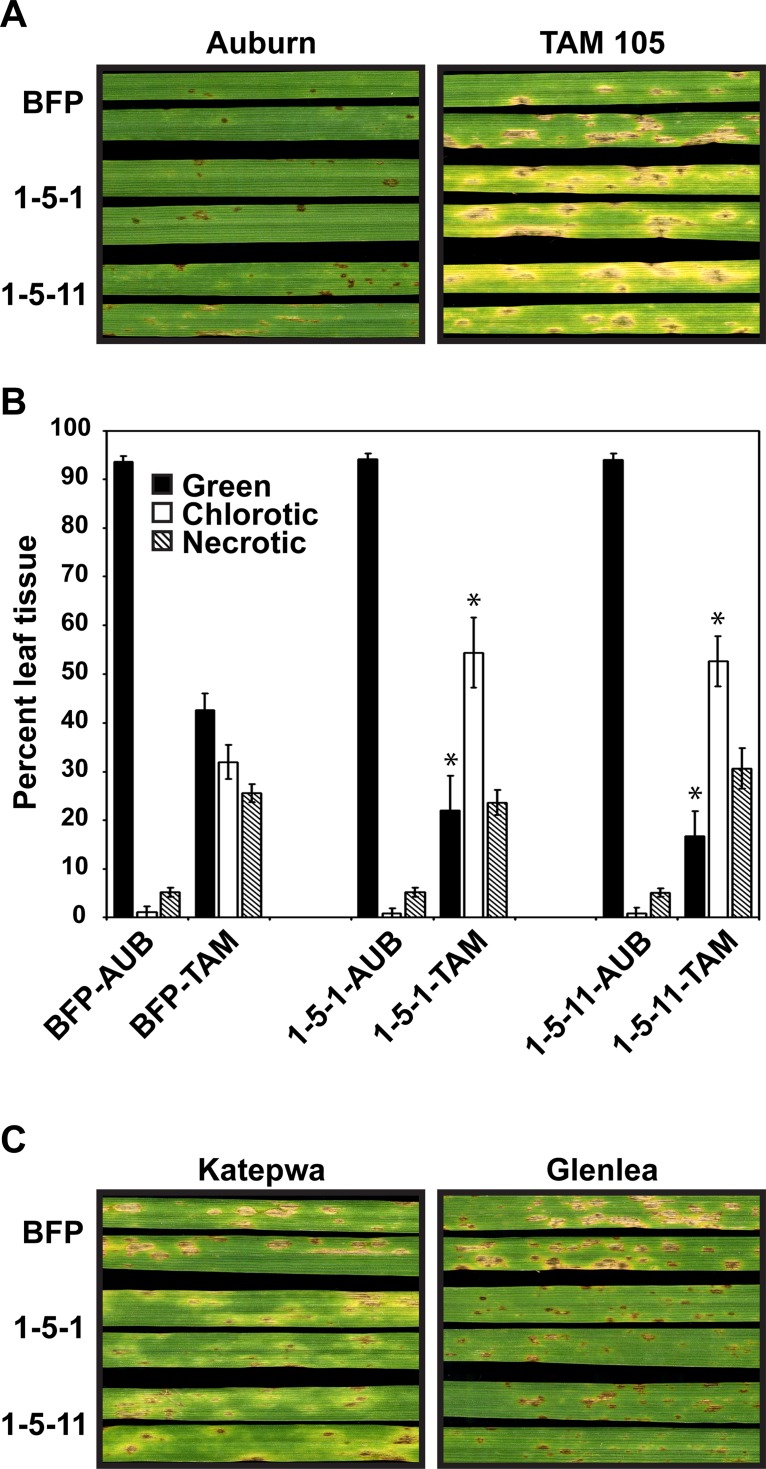
Symptom development induced by BFP and BFPΔ*toxA* isolates on ToxA-sensitive and -insensitive cultivars. The ToxA-insensitive and -sensitive cultivars, ‘Auburn’ and ‘TAM 105’, respectively, were inoculated and the symptoms (A) monitored and (B) quantified (chlorosis and necrosis). Bars represent means of values from three leaves per experiment from three independent experiments (nine total), error bars represent standard error, and * indicates a statistical difference from BFP as measured by a Student’s t-test (*P* < 0.05). (C) Symptom development on two additional ToxA-sensitive cultivars, ‘Katepwa’ and ‘Glenlea’. Leaves were harvested 5 days post inoculation.

We next tested whether disease development on ToxA-sensitive and a resistant cultivar (ToxA-insensitive) was impacted by *ToxA* replacement (Fig 3). Inoculation of the tan spot resistant cultivar, ‘Auburn’, by BFP, 1-5-1, and 1-5-11 resulted in a typical resistance reaction of a few small dark brown lesions with most of the leaf tissue remaining green (Fig 3A, left panel). This is in contrast to the reaction of the ToxA-sensitive cultivar ‘TAM 105’ with BFP, which showed symptom development that included the typical ToxA-induced tan-necrotic lesion with a dark center and green leaf tissue where the lesions did not coalesce (Fig 3A, right panel, top leaves). Inoculation of *ToxA*-knockout isolates, 1-5-1 and 1-5-11, on ‘TAM 105’ induced similar-sized tan-necrotic lesions with darkened centers, but also resulted in a spreading chlorosis symptom not seen in inoculations of the ToxA-expressing parent isolate, BFP (Fig 3A, right panel, middle and bottom leaves). By quantifying the percentage of green, chlorotic, and necrotic tissue, we found that the amounts were similar in all of the ‘Auburn’ inoculations; however, inoculation of *ToxA*-knockout isolates on ‘TAM 105’ resulted in less green tissue, more chlorotic tissue, and the same amount of necrotic tissue as BFP (Fig 3B). Therefore, inoculation of the ToxA-knockout isolates on ‘TAM 105’ actually resulted in greater leaf area affected by disease than inoculation of the ToxA-expressing parent isolate BFP.

Inoculation of BFP on the ToxA-sensitive cultivar ‘Katepwa’ also resulted in the typical ToxA-induced tan spot lesion (Fig 3C, left panel, top leaves); however, inoculation of the *ToxA*-knockout isolates produced lesions that contained small tan-necrotic centers surrounded by chlorosis, and spreading chlorosis throughout the leaf (Fig 3C, left panel, middle and bottom leaves), with the result of greater leaf area affected in ToxA-knockout inoculations than in BFP inoculations. In inoculations of a third ToxA-sensitive cultivar, ‘Glenlea’, again BFP induced typical tan-necrotic lesions, but the ToxA-knockout isolates appear to be reduced in the amount of disease produced, with small dark brown necrotic lesions being the major symptom. These data show that ToxA symptom development may be epistatic to other symptoms in some cultivars. On the ToxA-sensitive cultivar ‘TAM 105’, ToxA symptom expression was epistatic to the expression of symptoms induced by a toxin with similar necrosis-inducing activity as ToxA and a spreading chlorosis toxin. On ‘Katepwa’, ToxA-induced symptoms were epistatic to symptom expression caused by a toxin(s) that induces small necrotic lesions with associated chlorosis and spreading chlorosis. ‘Glenlea’ was the only ToxA-sensitive cultivar in which ToxA-induced epistasis was not apparent.

**Fig 4:**
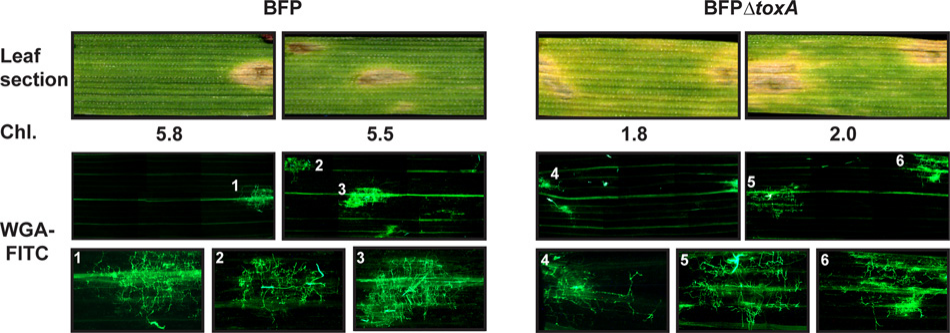
Mycelial growth *in planta* is restricted to lesions in BFP and BFPΔ*toxA* inoculated ‘TAM 105’. One centimeter leaf sections harvested from BFP and BFPΔ*toxA* inoculated leaves 6 days post inoculation were scanned (Leaf section), chlorophyll concentration estimated (Chl.), and mycelia stained with WGA-FITC. Stained leaves were imaged with a fluorescent microscope and the whole leaf image is a montage of 6 separate panels that cover the entire 1 cm leaf section. The bottom row represents increased magnification images of select (numbered) lesions.

It is possible that the increase in chlorosis with *ToxA*-knockout inoculations on ‘TAM 105’ was due to either a diffusible factor or proliferation of mycelia. To test this, we adapted the procedure of Ayliffe and colleagues [38] to stain 1 cm leaf sections with FITC-labeled wheat germ agglutinin (WGA-FITC), which binds to chitin, and visualized the stained leaf sections with fluorescent microscopy (Fig 4). Each stained section contained at least a single lesion (Fig 4, Leaf section). Estimation of total chlorophyll showed less chlorophyll in sections taken from leaves inoculated with *ToxA* knockouts compared to the ToxA-expressing parent isolate BFP (Fig 4, Chl.), consistent with the increase in spreading chlorosis in *ToxA* knockout-inoculated leaves (Fig 3). Mycelial staining was easily visualized in necrotic lesions only (Fig 4, WGA-FITC), and examination at higher magnifications confirmed that very little mycelial growth occurred outside of the large necrotic lesions (Fig 4, WGA-FITC, bottom row). Horizontal lines of fluorescence were associated with vascular tissue and a background haze of fluorescence was often associated with a lesion, but we considered this as non-WGA-FITC-associated fluorescence. These data suggest that the spreading chlorosis was not due to proliferation of mycelia but rather to a diffusible factor, possibly ToxC.

### Heterologous expression of *ToxA* in a *P*. *tritici-repentis* race 3 isolate

**Fig 5:**
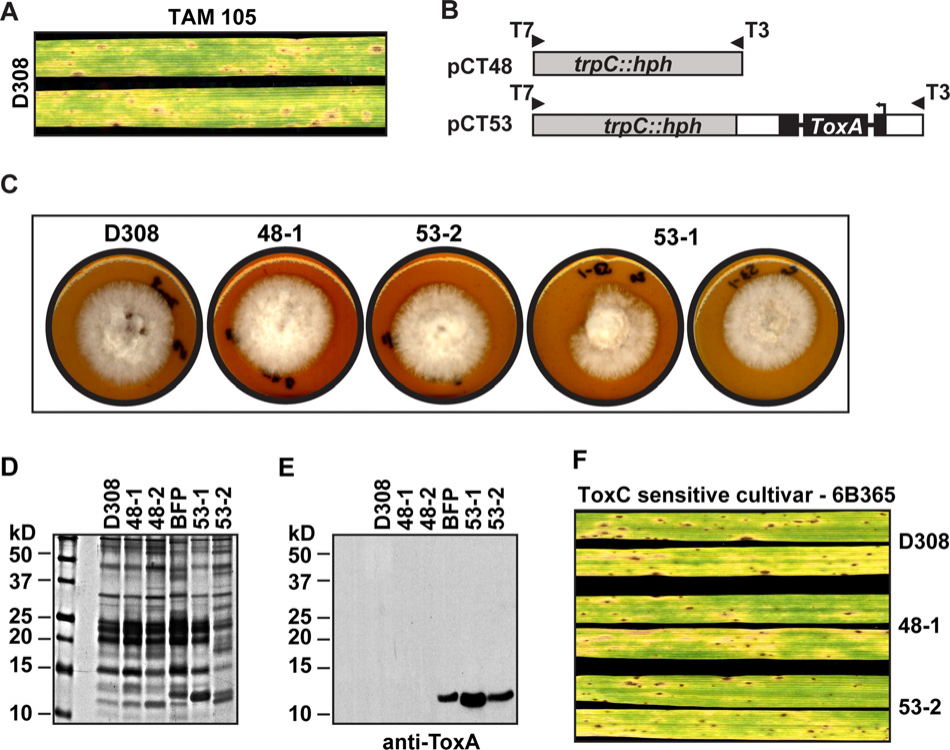
Characterization of growth and toxin production in D308 and D308-*ToxA* ^*+*^ isolates. (A) Inoculation of ‘TAM 105’ with a race 3, ToxC-producing isolate, D308. (B) Constructs used as templates for PCR amplification with T3 and T7 primers for DNA fragment generation for genetic transformation of D308. Plasmid constructs were originally described in [12]. (C) Colonial morphology of isolates grown for 6 days on V8-PDA agar. Note the abnormal morphology of 53–1. SDS-PAGE of equal amounts of protein from crude culture filtrate either (D) silver-stained or (E) on western blot with anti-ToxA antisera. The molecular mass of standards and their position are indicated on the left of each gel. (F) Inoculation of isolates on the ToxC-sensitive cultivar ‘6B365’. Leaves were harvested 6 days post inoculation.

As shown (Fig 2D), ToxC induces spreading chlorosis on the ToxC-sensitive cultivar ‘6B365’ that is similar to the spreading chlorosis produced on ‘TAM 105’ by BFPΔ*toxA* isolates (Fig 3A). However, to our knowledge it has not been shown that ‘TAM 105’ is ToxC-sensitive. To test this, we inoculated ‘TAM 105’ with the race 3 ToxC-producing isolate, D308. D308 is a slower-growing isolate than BFP that sometimes grows abnormally without radial symmetry on V8-PDA plates (0–1 abnormal colonies/5 total plates). Additionally, those colonies that grow abnormally do not conidiate as well as those with symmetric radial growth. Because of the reduced growth rate, we assessed symptom development at 6 dpi rather than 5 dpi as with BFP and its derived isolates. D308-induced symptoms on ‘TAM 105’ included small light brown to tan necrotic lesions, some with chlorotic haloes, and spreading chlorosis (Fig 5A), consistent with ‘TAM 105’ sensitivity to ToxC.

We next sought to determine if *ToxA* expression by D308 would inhibit the spreading chlorosis symptom on ‘TAM 105’ as was observed for the ToxA- and ToxC-producing race 1 BFP isolate. Therefore, we transformed D308 protoplasts with a *ToxA*-expression construct that includes *hygR* (*hygR*::*ToxA*) and a construct containing *hygR* alone as a control (Fig 5B) and generated two control (48–1 and 2) and two *ToxA*-transformed (53–1 and 2) *hygR* colonies in a single transformation. All transformants grew similarly to D308 on V8-PDA with the exception of 53–1, which grew more slowly and individual colonies more often had abnormal radial growth than the parent isolate (0–3 abnormal colonies/5 total V8-PDA plates; examples of colonial morphology presented in Fig 5C). To quantify differences in radial growth between D308 and 53–1 we compared colony size after 6 days of growth on V8-PDA (D308: 30.6 +/- 0.3 and 32.6 +/- 0.4 cm^2^ vs 53–1: 26.9 +/- 1.6 and 21.6 +/- 1.8 cm^2^) and found that growth of 53–1 was always slower and more variable than D308 (88 and 66% of D308). Whether the more irregular growth pattern of 53–1 was due to the insertion position of the transforming construct or over-expression of *hygR*::*ToxA* was not determined. To confirm expression of *ToxA* by the *hygR*::*ToxA* transformed isolates, we prepared CCF from D308, the control (48–1 and 2) and *ToxA*-transformed (53–1 and 2) isolates, and BFP as a ToxA-producing control, separated equal amounts of precipitated protein by SDS-PAGE, and detected proteins via silver stain and ToxA specifically by western blotting with anti-ToxA antisera. The silver-stained gel showed only BFP and the *ToxA*-transformed isolates, 53–1 and 53–2, with a band at ~ 13 kDa, consistent with ToxA production (Fig 5D). The amount of ToxA produced in CCF by BFP and 53–2 was comparable, whereas more was produced by 53–1. These observations were confirmed by western blotting (Fig 5E). We used D308, the control transformant 48–1, and the *ToxA* transformant 53–2 for further testing, as 53–2 produced similar levels of ToxA as the reference isolate BFP. All isolates induce small dark brown lesions and similar levels of chlorosis on the ToxC-sensitive cultivar ‘6B365’ (Fig 5F).

### Disease reaction and *in planta* mycelial growth of *ToxA*-transformed race 3 isolates

**Fig 6:**
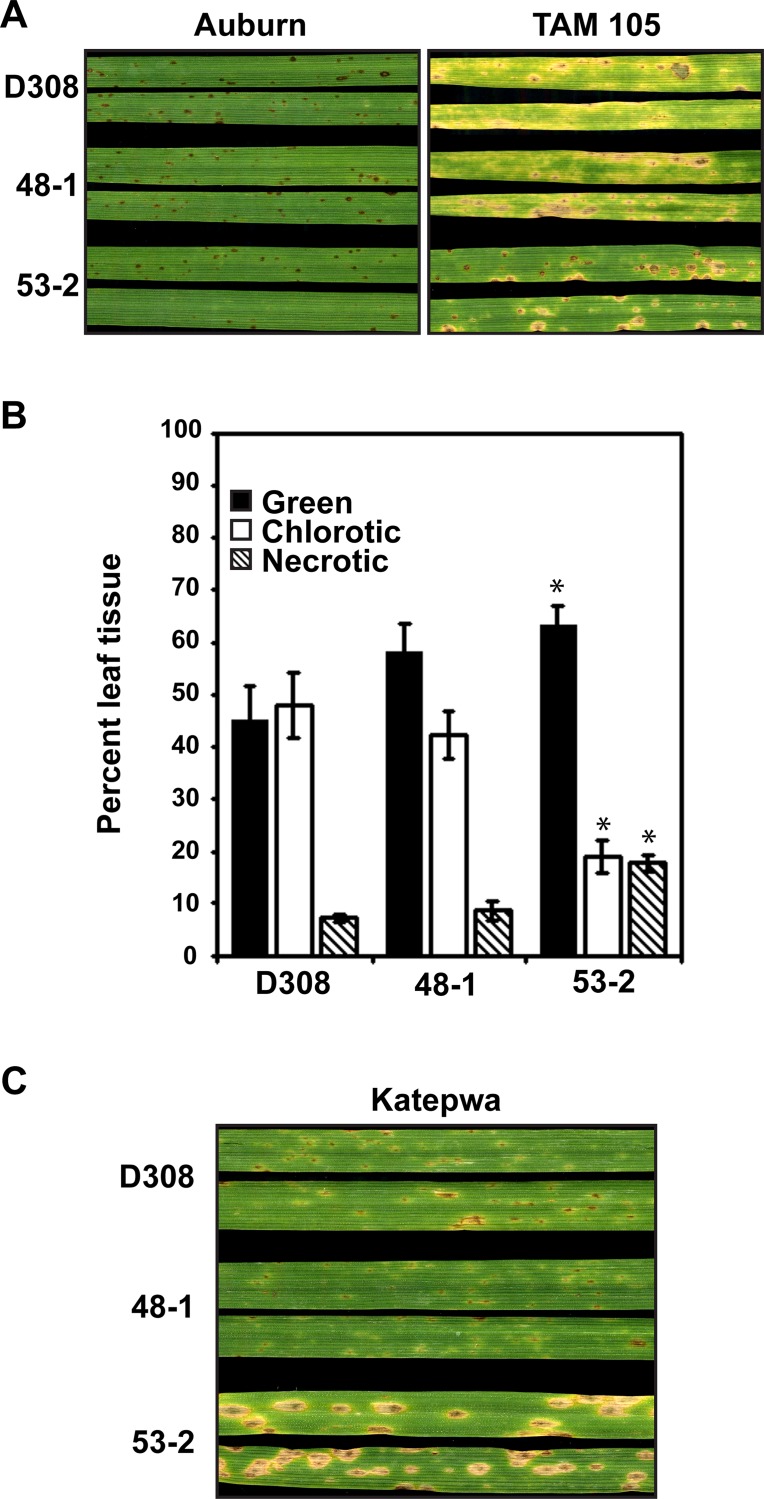
Symptom development induced by D308 and D308 transformants on ToxA-sensitive and -insensitive cultivars. The ToxA-insensitive and -sensitive cultivars, ‘Auburn’ and ‘TAM 105’, respectively, were inoculated and the symptoms (A) monitored and (B) symptoms on ‘TAM 105’ quantified (chlorosis and necrosis). Bars represent means of three leaves per experiment from three independent experiments (nine total), error bars represent standard error, and * indicates a statistical difference from D308 as measured by a Student’s t-test (*P* < 0.05). (C) Symptom development on the ToxA-sensitive cultivar, ‘Katepwa’. Leaves were harvested 6 days post inoculation.

Inoculation of ‘Auburn’ with D308 and the D308 transformants 48–1 and 53–2 produced a typical resistance reaction (Fig 6A, left panel). On ‘TAM 105’, spreading chlorosis was seen in inoculations of D308 and the *hygR* control, 48–1, but not in inoculations of the *ToxA*-expressing transformant, 53–2, where the symptom was primarily necrotic lesions (Fig 6A, right panel). Inoculations of 53–1 on ‘Auburn’ and ‘TAM105’ gave a similar phenotype to those of 53–2 on the same cultivars, but the necrotic lesions on ‘TAM105’ were larger, perhaps due to increased ToxA expression (S1 Fig). Quantification of the percent of green, chlorotic, and necrotic tissue shows that the *ToxA*-expressing transformant, 53–2, had more green and necrotic tissue, and less chlorotic tissue than D308 (Fig 6B), consistent with the ToxA epistasis of ToxC symptom development. Inoculation with D308 and 48–1 of the ToxA-sensitive cultivar ‘Katepwa’ showed very little disease development, but with 53–2 showed typical tan necrosis lesions induced by ToxA (Fig 6C).

**Fig 7:**
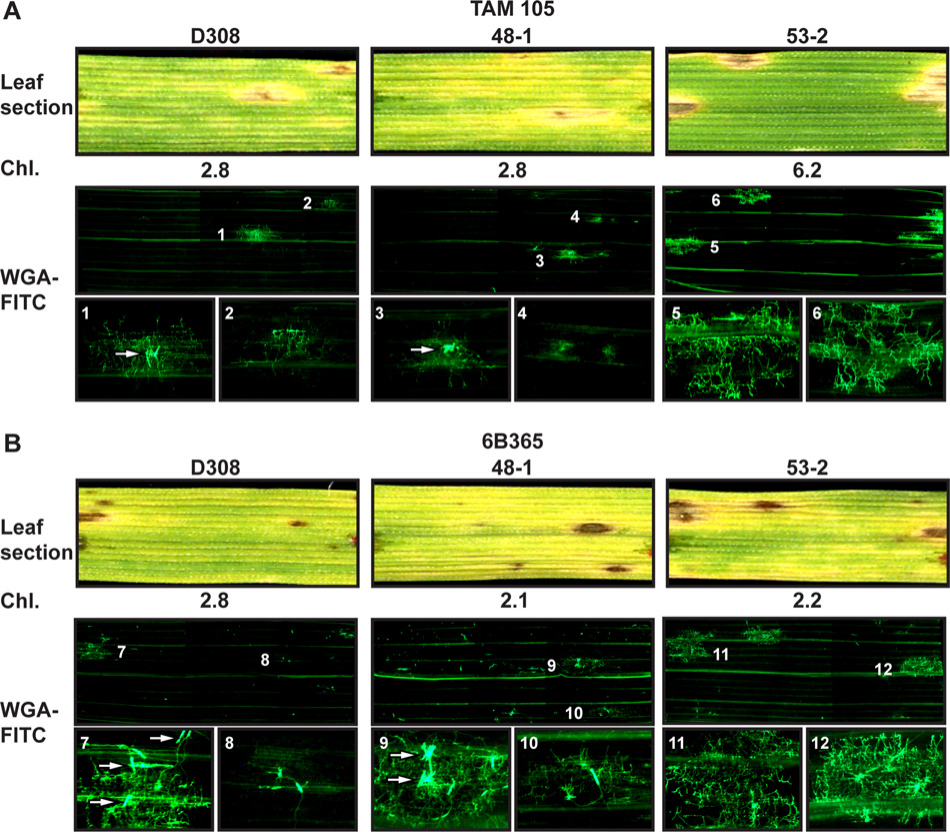
Mycelial growth *in planta* of D308 and D308 transformants. Isolates were inoculated on the ToxA- and ToxC-sensitive cultivars (A) ‘TAM 105’ and (B) ‘6B365’, respectively. One centimeter leaf sections harvested from inoculated leaves 6 days post inoculation were scanned (Leaf section), chlorophyll concentration estimated (Chl.), and mycelia stained with WGA-FITC. Stained leaves were imaged with a fluorescent microscope and the whole leaf image is a montage of 6 separate panels that cover the entire 1 cm leaf section. The bottom row represents increased magnification of the same size of select (numbered) lesions. Arrows point to multiple conidia.

To determine if the spreading chlorosis seen in D308 and 48–1 inoculated ‘TAM 105’ was due to a diffusible factor or mycelial growth as investigated in inoculations of the BFPΔ*toxA* isolates, we stained leaf sections of inoculated ‘TAM 105’ with WGA-FITC (Fig 7A). Estimation of total chlorophyll present in each leaf section of ‘TAM 105’ analyzed showed lower chlorophyll content in the D308- and 48-1-inoculated leaf sections compared with those of the *ToxA*-transformed isolate, 53–2 (Fig 7A, Chl.), consistent with the chlorotic symptoms induced by the non-ToxA expressing isolates. Significant mycelial staining was visualized only in lesions (Fig 7, WGA-FITC), and examination at higher magnifications confirmed that very little mycelial growth occurred outside of the lesions (Fig 7A, WGA-FITC, bottom row). Mycelial growth was much less in the D308- and 48-1-induced lesions when compared to the 53-2-induced lesions. In fact, some D308 and 48–1 lesions showed very little mycelial growth (Fig 7A, e.g. lesion 2) and an apparent lack of penetration (Fig 7A, e.g. lesion 4). As expected, expression of ToxA by the ToxA transformant, 53–2, provided a benefit for mycelial growth in the ToxA-sensitive ‘TAM 105’ cultivar (Fig 7A, compare lesions 5 and 6 with 1 through 4).

Given the above data, it seems likely that the spreading chlorosis in ‘TAM 105’ inoculations with ToxC-expressing isolates that lack ToxA is due to ToxC, and furthermore, that the chlorosis is due to ToxC diffusion and not mycelial growth throughout the tissue. To further evaluate this, we assessed mycelial growth in the ToxC-sensitive cultivar ‘6B365’ inoculated with D308, the control transformant 48–1, and the *ToxA*-expressing transformant, 53–2 (Fig 7B), all of which induce similar levels of spreading chlorosis in ‘6B365’ (Figs 5F and 7B). All leaf sections examined had small to medium-sized dark brown lesions and significant spreading chlorosis (Fig 7B, leaf section) as confirmed by the low chlorophyll content (Fig 7B, Chl.). Mycelial growth was confined to lesions (Fig 7B, WGA-FITC). In some lesions, particularly in leaves inoculated with D308 and 48–1, there was very little growth (Fig 7B, e.g. lesions 8 and 10) and the larger lesions that contained greater amounts of mycelia were often associated with multiple conidia (Fig 7B, arrows). In contrast, greater mycelial growth was typically evident in 53–2 lesions, and these were not necessarily associated with multiple conidia (Fig 7B, e.g. lesion 11). Thus, visual inspection of mycelial growth suggested that in ‘6B365’, 53–2 grows better than D308 and 48–1.

## Discussion

In this study, we utilize multiple methods for gene replacement in the genome of the tan spot pathogen, *P*. *tritici-repentis* (Fig 1). LME-mediated transformation produced many transformants, but only a small percentage of these were homologous integrants; therefore, although this method is very efficient in the closely-related species *Alternaria brassicicola* [39], it does not appear to be optimal for *Ptr*. Transformation with either large-linear or split-marker fragments produced a high percentage of gene-replacing recombinants. In this study, the large-linear fragment approach appears to have resulted in at least one recombinant with multiple fragments inserted, which suggests that the split-marker approach may be best for this organism/genome. A fusion PCR approach was also successfully used to generate a *ToxA* replacement construct for transformation of an Australian *Ptr* field isolate, although a different antibiotic was used for selection [41]. The ability to ‘knockout’ single and ‘silence’ multicopy genes [42,43] are important tools for studying tan spot as both single and multi-copy genes are known to impact virulence [44,45] and others are predicted to play a major role in disease establishment [33].

Gene replacement of *ToxA* in the race 1 *Ptr* isolate BFP resulted in no ToxA production and did not affect isolate growth or ToxC-induced spreading chlorosis in the ToxC-sensitive cultivar ‘6B365’ (Fig 2). Current (Fig 3) and previous work [12] from our group has shown that inoculations of BFP on ‘TAM 105’ produced only necrotic lesions, consistent with sensitivity to a necrosis-inducing toxin ([17] and (Fig 3)). Contrary to our expectations, in the absence of ToxA expression by BFP, the amount of necrosis produced in inoculations of ‘TAM 105’ was unchanged and the amount of total symptom development increased, with the appearance of spreading chlorosis (Fig 3). This indicates that expression/perception of the potent necrotizing toxin, ToxA, prevents visualization of symptoms induced by a chlorosis- and additional necrosis-inducing toxin(s). In other words, ToxA symptom development is epistatic in ‘TAM 105’. The appearance of additional symptoms is likely due to the recognition of additional toxins produced by this isolate, which may include (but may not be limited to) two putative, uncharacterized necrosis-inducing toxins and ToxC [17,33]. Although we cannot be certain that the spreading chlorosis observed in 'TAM 105' is due to ToxC, inoculation of ‘TAM 105’ with another known ToxC-producing isolate, D308, also induced spreading chlorosis, which was repressed in ToxA-expressing D308 transformants (Fig 5). Taken together, these data suggest that expression of ToxA can obscure the expression of symptoms induced by other toxins, at least in certain wheat genotypes.

Epistasis by ToxA appears to be cultivar dependent. Inoculations of BFP-ToxA knockouts on the ToxA-sensitive cultivar ‘Katepwa’ resulted in a previously undetectable spreading chlorosis and a reduced necrosis phenotype with extended chlorotic haloes (Fig 3), showing epistasis of ToxA to other toxins. However, ToxA epistasis is not apparent in inoculations of the cultivar ‘Glenlea’, where in the absence of ToxA, BFP induces only small necrotic lesions and disease severity is limited (Fig 3). Moffat and colleagues [41] also inoculated race1Δ*toxA* isolates on ‘Katepwa’ and ‘Glenlea’, with similar results, although the spreading chlorosis that we visualize in ‘Katewpa’ is not as evident and the extent of disease is reduced in their inoculations. The difference in the appearance of the spreading chlorosis could be because the isolates used in the two studies differ, and therefore are likely to have different suites of HSTs. It is also possible that inoculation conditions and/or the way plants were incubated post inoculation may have contributed to this difference, as it is known that ToxC-symptom development can be impacted by environmental conditions [26] and we have seen that it is highly dependent on the amount of light leaves are exposed to post-inoculation. The detection of additional symptoms in the absence of ToxA is consistent with evidence supporting the presence of additional toxins in race 1 isolates and their corresponding sensitivity loci in the host. In support of this, EMS-derived mutants of the ToxA-sensitive wheat cultivar ‘Kulm’ that are no longer ToxA-sensitive still develop necrotic lesions in response to a race 1 isolate, but in some inoculations these lesions are less delimited and more chlorotic [46], similar to the necrotic/chlorotic lesions in inoculations with BFPΔ*toxA* on ‘Katepwa’. What is very interesting in the light of findings reported here is that ‘TAM 105’ as well as other wheat cultivars have been shown to exhibit both necrosis and spreading chlorosis symptoms in response to various *Ptr* race 1 isolates [47,48]. Cultivar-dependent epistasis of ToxA, and possibly other toxins, reveals an additional challenge in the identification of new HSTs in the *Ptr*-wheat pathosystem. These challenges can be overcome with a greater understanding of the complement of HSTs present in a global population, the ability to heterologously express toxins in tox^-^ isolates, and screening of diverse host germplasm.

We propose several mechanisms that could explain ToxA epistasis. ToxA expression could cause a reduction in the expression of genes responsible for ToxC production. While not testable until more is known about the genes required for ToxC production, this is unlikely as ToxC-associated symptom development occurs in the ToxC sensitive cultivar ‘6B365’ inoculated with isolates and transformants that make ToxC regardless of whether or not they produce ToxA (Figs 2 and 5). Also, wheat cultivars exist that exhibit both ToxC and ToxA symptoms simultaneously [47]. It is more likely that genotype-specific plant responses to ToxA and possibly other, previously undetected toxins are responsible for this epistasis. In sensitive leaves, ToxA induces rapid transcriptional [31], proteomic [49], and metabolic changes [49,50] and leaf collapse and the onset of cell death occur within 14 h of treatment [32]. This is more rapid than the appearance of ToxC symptoms, which typically are not visible until 3–4 dpi (data not shown). Rapid plant cell death could prevent diffusion of ToxC or other, previously undetected toxic activities, for example by causing tissue desiccation or conversely, by signaling the fungus to stop production. Why this does not occur in the host genotypes that develop both necrosis and chlorosis in response to ToxA- and ToxC-expressing race 1 isolates is unclear, but suggests the possibility that the spreading chlorosis on these host is due to sensitivity to an as yet undescribed toxin. Another intriguing possibility is that triggering the R gene-like ToxA-sensitivity gene *Tsn1* in some genotypes leads to suppression of other resistance-like gene responses that may be necessary for the expression of additional HST-induced symptoms. This is not without precedence as it has been shown that interaction between the R gene *I* in *Solanum [Lycopersicon] pimpinellifolium* and *Avr1* in *Fusarium oxysporum* f.sp. *lycopersici* suppresses disease resistance mediated by *I*-2 and *I*-3 [51]. The ‘suppression’ signal would have to be diffusible and/or systemic, as the development of spreading chlorosis occurs in areas where there is no mycelial growth (Figs 4 and 7). The presence of a systemic signal is consistent with the induction of diffusible metabolites by ToxA in ToxA-sensitive hosts [50].

This current study also examines the association of symptom development and mycelial growth of *Ptr* throughout whole leaf sections, unlike the many elegant studies that have examined at higher magnification the germination, penetration, and *in planta* colonization of *Ptr* on both susceptible and resistant cultivars [35,52,53,54]. In this current work we confirm that mycelial growth within the plant leaf is typically restricted to lesions during early stages of disease, even when spreading chlorosis has affected almost the entire leaf section (Figs 4 and 7). In inoculations of D308 and its transformants some conidia germinate, do not penetrate, yet still develop small brown lesions (Fig 7). Whether non-penetrating mycelia or only those that have penetrated the leaf contribute to spreading chlorosis is not known. As expected, ToxA expression by D308-*ToxA* ^*+*^ results in an obvious increase in mycelial growth in the lesions produced on the ToxA-sensitive cultivar ‘TAM 105’. What was unexpected was that ToxA expression by D308 appears to contribute to a slight increase in mycelial growth in the ToxA-insensitive cultivar ‘6B365’ (Fig 7). If so, this would indicate that ToxA can act in a *Tsn1*-independent manner to provide an advantage to the fungus that allows for greater accumulation of mycelia. Additional studies as to whether such beneficial effects of *ToxA* expression in ToxA-insensitive hosts are a general phenomenon, or isolate dependent, wheat genotype dependent, or both are required to confirm these preliminary observations. Interestingly, though ToxC causes extensive leaf damage, *Ptr* isolates that express ToxC alone are not commonly found in infected wheat fields; rather, race 1 isolates that express both ToxA and ToxC are most commonly isolated [55,56,57,58,59]. Additional functions for HSTs in the absence of a sensitivity locus in the plant seem likely. For instance, our laboratory and others have shown that internal expression of *ToxA* in monocot and dicot hosts can lead to cell death in the absence of *Tsn1* [60,61]. Additionally, it has been shown that transcript abundance of the HST ToxB is positively correlated with the number and rate of production of appressoria on both sensitive and insensitive hosts [42,62], suggesting a role for ToxB in basic pathogen fitness. Also, recent work has indicated that necrotrophic pathogens must overcome plant defenses in order to establish infection [50]. For example, the HST victorin can enhance virulence of a biotroph in the absence of recognition by its cognate R gene [63]. The role of HSTs in virulence in the absence of their cognate plant recognition partners, perhaps during an abbreviated biotrophic phase, has thus far been underexplored, and the *Ptr*-wheat pathosystem provides a compelling model for this line of inquiry.

## Supporting Information

S1 Fig Symptom development induced by D308 transformant 53–1 on ToxA-sensitive and -insensitive cultivars. ToxA-insensitive cultivars ‘6B365’ and ‘Auburn’ and the ToxA-sensitive cultivar ‘TAM 105’ inoculated with the 53–1. Leaves were harvested 6 days post inoculation.
(TIF)

S1 Table Primers used for construction of the template for the *ToxA* replacement construct and screening of putative homologous recombinants. (DOCX)
