# Supplementary figures and images for: Necrotrophic Effector Epistasis in the Pyrenophora tritici-repentis-Wheat Interaction
Source: PLoS One. 2015 Apr 6;10(4):e0123548. doi: 10.1371/journal.pone.0123548 (PMC4386829; doi:10.1371/journal.pone.0123548)

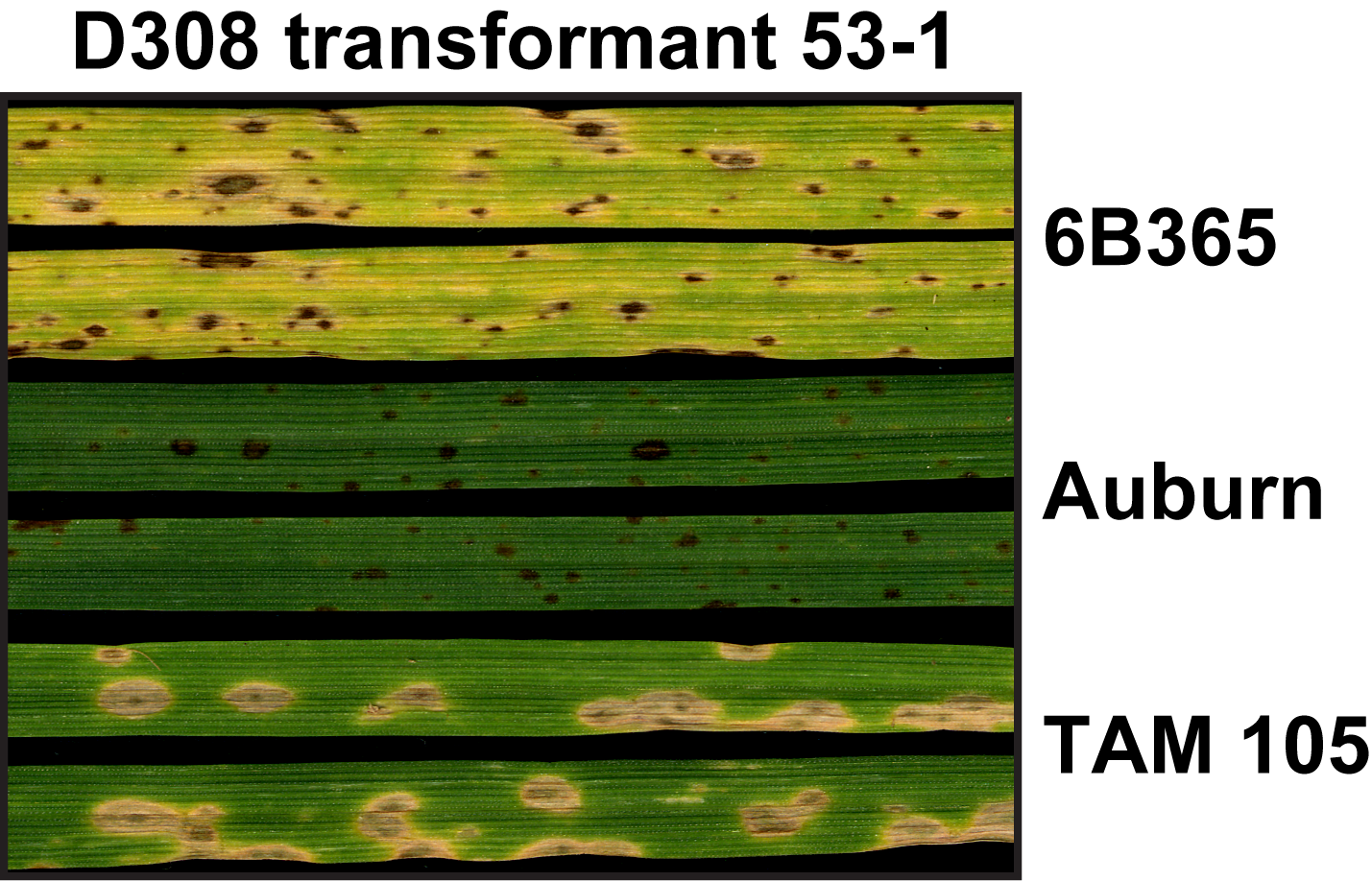

Supplement: S1 Fig — ToxA-insensitive cultivars ‘6B365’ and ‘Auburn’ and the ToxA-sensitive cultivar ‘TAM 105’ inoculated with the 53–1. Leaves were harvested 6 days post inoculation. (TIF) [file pone.0123548.s001.tif]
