# Supplementary material for: Necrotrophic Effector Epistasis in the Pyrenophora tritici-repentis-Wheat Interaction
Source: PLoS One. 2015 Apr 6;10(4):e0123548. doi: 10.1371/journal.pone.0123548 (PMC4386829; doi:10.1371/journal.pone.0123548)
Supplement: S1 Table — (DOCX) [file pone.0123548.s002.docx]

**Supplemental Table 1. Primers used for construction of the template for the *ToxA* replacement construct and screening of putative homologous recombinants.**

| Primer name | Primer start on supercontig 1.4^a^ | Sequence 5' to 3'^b^ |
| --- | --- | --- |
| TA-replacement-F1 | 1447368 | TACCACGATGTCACGCACAA |
| 5'flank-F1 | 1447600 | TA**GGGCCC**ATACAGTCCGTCCC |
| 5'flank-R1 | 1448600 | AG**CTCGAG**ACTTCGGAACCGACTCG |
| 3'flank-F1 | 1449809 | T**CCCGGG**CCCTCCCTTGCCATCCCTTG |
| 3'flank-R1 | 1450874 | T**GCGGCCGC**TTATAGCAGCATGGCGTAAG |
| TA-Replacement-R1 | 1451012 | TTGGAGACTTCAACCCGACG |
| TAKO-Split-F1 | N/A | CCCATACAGTCCGTCCCTTG |
| TAKO-Split-R1 | N/A | GAGGGCGTGGATATGTCCTG |
| TAKO-Split-F2 | N/A | TCGGTTTCAGGCAGGTCTTG |
| TAKO-Split-R2 | N/A | ATAGCAGCATGGCGTAAGCA |

^a^ Refers to supercontig of the BFP reference genome.

^b^ Letters in bold represent restriction endonuclease sites used in subcloning.
